# Factors which impact the length of hospitalisation and death rate of COVID-19 patients based on initial triage using capillary blood gas tests: a single centre study

**DOI:** 10.1038/s41598-022-22388-6

**Published:** 2022-10-19

**Authors:** Tomasz Ilczak, Alicja Micor, Wioletta Waksmańska, Rafał Bobiński, Marek Kawecki

**Affiliations:** 1 Department of Emergency Medicine, Faculty of Health Sciences, University of Bielsko-Biala, Willowa 2, 43-309 Bielsko-Biała, Poland; 2 European Pre-Hospital Research Network, Nottingham, United Kingdom; 3 Pulmonology and Thoracic Surgery Centre in Bystra, Bystra, Poland; 4 Department of Public Health, Faculty of Health Sciences, University of Bielsko-Biala, Bielsko-Biała, Poland; 5 Department of Biochemistry and Molecular Biology, Faculty of Health Sciences, University of Bielsko-Biala, Bielsko-Biała, Poland

**Keywords:** Risk factors, Predictive markers

## Abstract

An important element in the effective treatment of patients infected with the SARS-CoV-2 virus during the pandemic is an effective early triage to determine patient allocation and in-patient therapy. This paper assesses the prognostic value of capillary blood gas tests in predicting extended hospitalisation and death due to COVID-19. This retrospective statistical research is based on a group of 200 patients, hospitalised from 15 October 2020 to 08 March 2021. The study utilised the treatment documentation of these patients hospitalised due to COVID-19 at the Pulmonology and Thoracic Surgery Centre in Bystra (Southern Poland) during this period. The hospital has 50 beds with access to oxygen for COVID-19 patients and a five-bed intensive care unit. On the basis of the obtained results, conclusions were drawn that the need for early oxygen therapy with an oxygen mask and low pH values in capillary blood are significant risk factors for prolonging hospitalisation due to COVID-19. Age, the need for early oxygen mask therapy and low oxygen saturation are important risk factors for death from COVID-19. Capillary blood gas analysis is a simple and effective method of early in-patient segregation of COVID-19 patients.

## Introduction

The COVID-19 pandemic, which has been spreading around the world since 2019, continues to have a very dramatic trajectory. It has a direct influence on the functioning of health care systems all over the world^1^. Many medical procedures related to patient management have changed with most hospital emergency departments working under strict sanitary regimes or being completely converted to treat patients with confirmed COVID-19. The possibilities of diagnosing and confirming the presence of the virus at this point are very varied, from rapid antigen tests to PCR tests to in-hospital imaging diagnostics^2^. These in-hospital diagnostic possibilities, however, are very limited due to the very high infectivity^3^. The initial assessment of the patient is based on the presence of characteristic clinical symptoms such as a cough, increased body temperature and dyspnoea^4^. The main diagnostic problem is that the characteristic symptoms do not always indicate a need for hospitalisation, increased medical supervision or high-flow oxygen therapy. However, in some cases, even mildly symptomatic COVID-19 evolves into acute respiratory failure or multi-organ failure, where rapid intensive therapy and respiratory support are required^5^. It is very important to qualify the patient for appropriate treatment at the first stages of diagnosis and to identify the risk factors for severe COVID-19. A significant clinical problem, depending on the severity of the virus, is the period of hospitalisation, which may be prolonged depending on the complexity of the symptoms. Working conditions for medical staff during the pandemic have been very difficult—the amount of work and the stress associated with limitations on the equipment available^6^. Some procedures, such as imaging diagnostics or laboratory tests, are not possible when the patient arrives at the hospital. Correct, prompt assessment and qualification of the patient is crucial as it affects the functioning of the entire healthcare system. Hospital places are very limited and the correct allocation of the patient plays a key role for the patient and the entire health care system^7,8^. The solution to the problem of proper patient qualification together with early risk assessment, the prolongation of hospitalisation and possible death is to determine clinically significant predictors during the first examination and triage^9^. Every time a patient arrives at Accident and Emergency (A&E) they are interviewed and basic parameter measurements are performed, such as heart monitoring, blood pressure, temperature and blood glucose levels. Rarely is a capillary blood gas test performed on patients during triage^10^. Capillary blood testing takes only a little time and can be performed by anyone working in the system. Collecting capillary blood is within the competency of emergency nurses and paramedics, and the correct assessment of parameters does not require a consultation with a doctor.

This study, will endeavour to answer the question as to whether conducting an analysis of capillary blood parameters during triage can contribute to the early determination of predictors of hospitalisation time and death in a group of COVID-19 patients.

## Material and methods

The study was retrospective and covered the period from 15 October 2020 to 08 March 2021. The study utilised the documentation of patients who had been hospitalised at the Pulmonology and Thoracic Surgery Centre in Bystra (Southern Poland). The hospital offered 50 beds with oxygen for patients with COVID 19 and a five-bed intensive care unit.

### Analysis of documentation

The analysis of the documentation included the examination card, which is completed each time a patient is assessed in the emergency department. The observation card contains information on demographic features such as age, gender, medical history, and smoking habits. Basic parameters such as blood pressure, ECG monitoring and blood saturation are also collected. Data on the oxygen therapy used and its form are included, as well as the parameters of capillary blood gas tests performed on each patient at the time of their admission to the hospital. Other documentation collected for the purpose of conducting the study included cards detailing the in-hospital course of treatment, with particular emphasis on the length of hospitalisation and patients’ deaths.

In order to correctly select the material for the study group, a detailed documentation analysis plan was prepared and the criteria for including and excluding documentation from the study were applied.

The inclusion criteria included documentation of only those patients in whom COVID-19 was confirmed by a previous PCR test, where the initial triage took place in the emergency department, and the documentation had been completed at each stage of the hospitalisation. The analysis included documentation of those patients who had either not been given oxygen therapy directly before the capillary blood gas measurement or, if so, had only been given nasal cannulas and where this was clearly indicated on the patient evaluation card. The criteria for excluding material from the study included the documentation of patients who, after the initial triage, were sent home or transferred to another hospital, whose documentation was incomplete or who had died in the emergency department prior to admission to the ward. Documents of patients who, due to their very serious condition, were immediately transported to a ward, therefore bypassing the emergency department, were also excluded.

During the research period, 536 patients were seen at A&E and, after applying the inclusion and exclusion criteria, the documentation of 200 of them qualified for the next part of the study. The demographic characteristics of the study group are presented in Table 1. All collected data were correctly encoded to prevent identification. Immediately afterwards this, the information was transferred in the correct way to a specially prepared matrix in Microsoft Excel 2019 and analysed statistically. The Research Ethics Committee at the University of Bielsko-Biała has decided that this type of research meets the ethical criteria and does not require additional permission. No. 2022/1/9E/10.

**Table 1:** Demographic features of the studied group.

	Frequency	Percentage
**Gender**		
Female	89	44.50%
Male	111	55.50%
**Co-morbidities**		
Yes	150	75.00%
No	50	25.00%
**Smoker**		
Yes	28	14.00%
No	172	86.00%
**Death**		
Yes	52	26.00%
No	148	74.00%
**Oxygen**		
Yes	135	67.50%
No	65	32.50%
**pH**		
< 7.35	18	9.00%
7.35–7.45	83	41.50%
> 7.45	99	49.50%
**pCO2**		
< 35 mmHg	144	72.00%
35–45 mmHg	46	23.00%
> mmHg	10	5.00%
**pO2**		
< 70 mmHg	174	87.00%
70–100 mmHg	26	13.00%
**SaO2**		
< 94%	165	82.50%
94–98%	32	16.00%
> 98%	3	1.50%

### The procedure

At the patient’s first contact with the A&E staff, the standard procedure is for basic triage to be performed by the duty nurse or paramedic. The patient's documentation is completed and a medical history is collected. At the next stage, basic vital parameters are taken including capillary blood gas measurements. The only treatment that can be used without a doctor's authority is oxygen via an oxygen mask. Capillary blood gas testing is performed by trained personnel working in A&E. The diagnostic device is a Siemens Rapid Point 500 (serial no. 21-46105) and the collection procedure includes, according to the hospital standards, a puncture in the earlobe and collection of the blood into a heparinised capillary for analysis. Based on this analysis, decisions are made regarding the further treatment of the patient.

### Research hypothesis

Two research hypotheses were applied, which assume that certain parameters that can be obtained during triage may be predictors of the length of hospitalisation and death due to COVID-19. The parameters used for the evaluation during the study were the medical history indicating co-morbidities such as arterial hypertension, diabetes, coronary artery disease, asthma and chronic obstructive pulmonary disease (COPD) as well as smoking habits, capillary gas parameters such as pH, blood saturation (Sao2), partial pressure of oxygen (PaO2) and partial pressure of carbon dioxide (PaCo2), and the need or not for oxygen therapy with an oxygen mask on arrival at A&E.

### Statistical analysis

In the statistical study, α = 0.05 was adopted as the basic level of significance. A linear regression model was used to verify the assumed hypothesis regarding the factors influenciating the hospital stay and the R^2^ (McFadden) coefficient was interpreted. The assumption of normality of the distribution of the explanatory variable and the assumption of collinearity of the explanatory variables have been met. In order to assess the length of hospitalization, linear regression was used, and, after determining the significant factors, the marginal mean values were analysed. In the case of linear regression, the confidence interval was determined for the marginal means of the length of hospitalisation In order to verify the hypothesis that certain tested parameters significantly affect the possibility of death, a logistic regression model with a 95% confidence interval was used, where in the first part of the study the specified value of the good fit R2 coefficient was determined. The calculations were made in the R version 3.6.0 programming language, R libraries: jmv, Hmisc, ggplot2, tidyr PSPP version 20200905 daily, GPL v.3 licence and MS Office 2019.

### Ethics approval

The Research Ethics Committee at the University of Bielsko-Biała has decided that this type of research meets the ethical criteria and does not require additional permission. No. 2022/1/9E/10.

## Results

In Table 2, descriptive statistics such as the mean, minimum and maximum values, as well as the median values of variables such as age, number of days of hospitalisation and the results of capillary blood gas tests are shown.

**Table 2:** Descriptive statistics of the studied group.

	*N*	*M*	*SD*	*Min*	*Max*	*Me*
Age	200	67.17	13.82	22.00	93.00	67.50
Hospitalisation	200	13.94	7.55	1.00	39.00	12.00
pH	200	7.44	0.08	7.12	7.90	7.45
pCO2	200	32.99	7.85	17.30	87.10	32.10
pO2	200	55.88	12.07	17.10	92.70	54.85
SaO2	200	86.58	9.16	30.00	98.50	88.95

*N*—number; *M*—mean; *SD*—standard deviation; *Min*—minimum; *Max*—maximum; *Me*—median.

The detailed analysis presented in Table 3 shows that the necessity to use oxygen is a statistically significant influence on the hospitalisation period; the coefficient β = 0.39 indicates a moderately strong, positive dependence of the period of hospitalisation on the administration of oxygen. People who required oxygen therapy at the time of triage were hospitalised significantly longer. In addition, a significant influence of pH on the hospitalisation period was demonstrated with a coefficient of β =  − 0.19. This shows the presence of a weak and negative dependence of the hospitalisation period on the pH value. The higher the pH in the study group, the significantly shorter the hospitalisation time.

**Table 3:** Predictive factors for extending the duration of hospitalisation in patients diagnosed with COVID-19.

Explanatory variable	Model				Regression factors			
	*R*^*2*^	*F*	*df*	*p*	Predictor	*β*	*t*	*p*
Length of hospitalisation	0.05	2.12	190	0.030*	Constant		2.64	0.009**
					Gender : M–F	0.09	0.63	0.531
					Co-morbidities: yes–no	0.20	1.11	0.268
					Smoker: yes–no	− 0.25	− 1.17	0.245
					Oxygen therapy needed: yes–no	0.39	2.49	0.014*
					Age	− 0.13	− 1.57	0.119
					pH	− 0.19	− 2.51	0.013*
					pCO2	0.01	0.09	0.931
					pO2	− 0.23	− 1.78	0.077
					SaO2	0.18	1.40	0.163

*R*^2^—coefficient for model fit; *F*—test statistic; *df*—degrees of freedom; *β*—standardised coefficient beta; *t*—test statistic; *p*—statistical significance; *p < 0.05; **p < 0.01; ***p < 0.001.

The pH values presented in the Table 4 represent the assumed reference level of 7.44, which is the mean value of the test group and the limit values decreased and increased by the standard deviation. The reference level, i.e. 100%, was taken as the hospitalisation time of patients who did not require oxygen therapy and the mean marginal value of their pH was 7.44. Thus, the hospitalisation time of 10.88 days was assumed as 100%. When the pH was reduced to a mean value of 7.36 in patients who did not require oxygen therapy, the mean hospitalisation time was 12.29 days, an increase of 13.0% from the reference value. Increasing the mean marginal value of pH to 7.52 in patients who did not require oxygen therapy resulted in a reduction of hospitalisation time to 9.48 days, i.e. 12.9% from the reference time. In patients requiring oxygen therapy with the assumed mean marginal pH of 7.36, the hospitalisation time was 15.20 days and was 39.7% longer than the reference time. In patients who required oxygen therapy with an average marginal pH of 7.44, hospitalisation was extended to 13.80 days, i.e. 26.8%. In patients requiring oxygen therapy at pH values 7.52, the hospitalisation time was 12.39 days and was longer than the reference level by 13.9%.

**Table 4:** Marginal mean for the hospitalisation period depending on the pH value and the need for oxygen therapy.

Oxygen therapy	pH		*M*	*SE*	95% Confidence interval	
					Lower limit of the range	Higher limit of the range
No	7.36	^−^*1SD*	12.29	1.26	9.81	14.76
	7.44	^μ^*Mean*	10.88	1.14	8.63	13.14
	7.52	^+^*1SD*	9.48	1.29	6.93	12.03
Yes	7.36	^*−*^*1SD*	15.20	1.14	12.96	17.44
	7.44	^μ^*Mean*	13.80	1.00	11.82	15.78
	7.52	^+^*1SD*	12.39	1.16	10.10	14.69

*M*—mean, *SE*—standard error, ^−^mean − 1*SD*, ^μ^mean, ^+^mean + 1*SD.*

The detailed analysis presented in Table 5 shows the statistically significant influence of the need to administer oxygen, age and oxygen saturation on the occurrence of deaths. The assessment coefficient for the necessity to use oxygen therapy was positive, and by analysing the odds ratio it was shown that with the change of the variable value from ‘no need to use oxygen’ (no) to ‘need to use oxygen’ (yes), the probability of death increases 3.5 times. Age assessment was also positive, and the odds ratio shows that as age increases by 1 year, the likelihood of death increases by 7%. In the case of oxygen saturation of the blood, the coefficient was negative, and the odds ratio shows that as the SaO2 value increases by 1 point, the probability of death decreases by 8%.

**Table 5:** Predictors of death in patients diagnosed with COVID-19.

Regression coefficients, *χ*^2^(9, *N* = 200) = 60.27; *p* < 0.001; *R*^2^ = 0.26					95% Confidence interval	
Predictor	*B*	*W*	*p*	*exp(B)–OR*	Lower limit of the range	Higher limit of the range
Constant	8.67	0.44	0.662	5851.74	0.00	4.736*10^20^
Gender: M–F	0.1	0.25	0.799	1.11	0.51	2.41
Co-morbidity: yes–no	0.41	0.71	0.477	1.51	0.48	4.71
Smoker: yes–no	− 0.8	− 1.13	0.257	0.45	0.11	1.79
Oxygen therapy yes–no	1.51	2.78	0.005**	4.5	1.56	13.03
Age	0.07	3.29	0.001**	1.07	1.03	1.11
pH	− 0.92	− 0.35	0.727	0.4	0.00	68.25
pCO2	− 0.05	− 1.67	0.095	0.95	0.90	1.01
pO2	− 0.01	− 0.17	0.864	0.99	0.93	1.06
SaO2	− 0.08	− 1.99	0.046*	0.92	0.85	1.00

*R*^2^—coefficient for model fit; *χ*^2^—test statistic; *df*—degrees of freedom; *B*—estimating factor; *W*—test statistic; *p*—statistical significance; *exp(B)*—*OR—*odds ratio; *p < 0.05; **p < 0.01; ***p < 0.001.

## Discussion

Predictive factors in emergency procedures and triage are very important for every patient where there is a deterioration in health or a threat to life. During the COVID-19 pandemic, they have played an important role because in many emergency procedures time is the main determinant of patient survival. Nowadays, health care systems around the world are becoming ineffective due to the growing wave of patients diagnosed with COVID-19. Wang D^11^ in his study indicates that the median time of admitting a patient to the intensive care unit from the onset of symptoms is eight days. For this reason it is so important to quickly determine the prognosis for the patient and make the correct decisions with regard to the place of hospitalisation and the treatment to be used.

In this study, the effect of triage parameters combined with capillary gas analysis in a group of COVID-19 patients was assessed. There is nothing published in the available literature about the risk factors contributing to a prolonged stay in hospital and most researchers focus on the risk of death or the parameters indicating the need for hospitalisation in ICU^12–14^. The research conducted so far has not covered the issues linking the need for oxygen supply with the prediction of hospitalisation time and mortality. In most cases, patients hospitalised with COVID-19 will require oxygen therapy, but this is not always needed at the time of admission to the hospital. The reasons why patients with COVID-19 are admitted to hospital wards are not always directly related to respiratory failure, and the course of the disease may lead to the occurrence of conditions not yet specified in the pathophysiology of other diseases^15^. In one of the hypotheses of this particular study, it was assumed that the need for early oxygen therapy with an oxygen mask would be a potential risk factor for prolonged treatment and death. The statistical analysis performed showed that patients who require immediate oxygen therapy on admission to the hospital are hospitalised significantly (*p* = 0.014) longer. Another statistically significant predictor influencing the prolongation of hospitalisation is the pH value measured in capillary blood (*p* = 0.013). There are no studies on this subject in the available literature and most of the publications on blood gas analysis are based on arterial gas test measurements^16^. This is surprising because despite the lower specificity of capillary gas tests, especially during a pandemic, it is much faster to perform and is a more accessible diagnostic method. In most cases, the analysis of blood gas parameters, including blood pH, is treated only as an indication for mechanical ventilation or high-flow oxygen therapy^17^. In the available studies, only Bezuidenhout MC^18^ associated the pH value with COVID-19 survival rates and showed that those patients with higher pH values had a greater survival rate. In his study, the correlation of the PaO2 value with survival was also proven, which is not covered in this research and may result from the fact that the PaO2 level is not adequate in capillary and arterial blood. In a study on a group of Italian patients, Turcato et al.^19^ showed that in the majority of patients admitted due to respiratory failure and pulmonary inflammation, the pH parameters were elevated. This is not normal because acidosis is usually a disorder in patients admitted to hospital due to respiratory failure^20^. The authors of the above studies did not attempt to correlate this parameter with the elongation of hospitalisation time. In this research, no attempts were made to assess the patient's condition in terms of acidosis or alkalosis, but only the form of the prediction of the length of hospitalisation based on the pH value was determined. The results clearly indicate that patients who, upon admission to the hospital, had higher capillary pH values required significantly shorter hospitalisation. Further analysis showed that a lower pH, along with the need to administer oxygen therapy with an oxygen mask at the time of admission, are the factors influencing a prolonged COVID-19 hospital stay by 39.7%. In the next stage of the study, the analysed parameters were assessed in the context of the prognosis of death. There are many publications describing the factors influencing the worsening of the patient's prognosis. Wang et al.^12^ and Berenguer et al.^13^ showed that low saturation values are a predictive factor for admitting patients to the intensive care unit. Lancet et al.^21^ in his study indicates that patients with a pre-hospital SpO2 below 90% much more often require hospitalisation, and in their studies Dillon et al.^22^ and Wang et al.^23^ associated low saturation values with patient death due to COVID-19. In this research, it was shown that a 1% increase in saturation statistically significantly (*p* = 0.046) reduces the probability of death due to COVID-19, which is consistent with previous studies. Other studies on the risk factors associated with the occurrence of death due to COVID-19 indicate that age and male gender are predictors of death. Lakbar et al.^24^ proved in their study that the probability of death due to COVID-19 in a group of men is higher than in a group of women, which was not confirmed in this study group. Chen et al.^25^ indicate that the elderly are more likely to die due to COVID-19 and the results obtained in our study show that age statistically significantly (*p* = 0.001) increases the risk of death due to COVID-19. Numerous studies on predictors of death in a patient with COVID-19 have shown that the presence of co-morbidities has a direct impact on the increase in mortality in a group of patients with COVID-19^26–28^. In the studied group, the presence of co-morbidities such as hypertension, diabetes, coronary artery disease, asthma, COPD was not a significant risk factor for death.

### Limitations of the study

The main limitation of the study is the lack of external validation of the research model. The authors of this study realise that in order to fully verify the assumed research hypotheses, it is necessary to evaluate the model. The study was conducted retrospectively and not all data were complete which resulted in a high number of rejections of the study material. To fully corroborate research model, multi-centre studies are needed.

## Conclusions

1. The need for early oxygen therapy using an oxygen mask and low pH values in capillary blood are significant risk factors for prolonging hospitalisation due to COVID-19.
2. Age, the need for early oxygen therapy with an oxygen mask and low oxygen saturation are significant risk factors for death due to COVID-19.
3. Capillary blood gas analysis is a simple and effective method of early segregation of hospital patients with COVID-19.

## Data Availability

The datasets generated during and/or analysed during the current study are available from the corresponding author on reasonable request.
